# Comprehensive analysis of transcriptome characteristics and identification of *TLK2* as a potential biomarker in dermatofibrosarcoma protuberans

**DOI:** 10.3389/fgene.2022.926282

**Published:** 2022-09-05

**Authors:** Xiao Zhang, Di Sun, Haiyan Zheng, Yamin Rao, Yuqi Deng, Xiao Liang, Jun chen, Jun Yang

**Affiliations:** ^1^ Department of Plastic and Reconstructive Surgery, Shanghai Ninth People’s Hospital, Shanghai Jiao Tong University School of Medicine, Shanghai, China; ^2^ Department of Pathology, Shanghai Ninth People’s Hospital, Affiliated to Shanghai Jiaotong University School of Medicine, Shanghai, China; ^3^ Department of Dermatology, Shanghai Ninth People’s Hospital, Affiliated to Shanghai Jiaotong University School of Medicine, Shanghai, China

**Keywords:** dermatofibrosarcoma protuberans, RNA sequencing, Tlk2, fusion genes, tumor microenvironment

## Abstract

**Background:** Dermatofibrosarcoma protuberans (DFSP) is a rare cutaneous sarcoma characterized by local invasion and recurrence. RNA sequencing (RNA-seq) allows the qualification of cellular RNA populations and provides information on the transcriptional state. However, few studies have comprehensively analyzed DFSP transcriptional data.

**Methods:** Fourteen DFSP samples with paired non-neoplastic soft tissue from Chinese patients undergoing Mohs micrographic surgery were used for RNA-seq analysis. Differential expression analysis and enrichment analysis for RNA-seq data were performed to identify fusion genes, biomarkers, and microenvironment characteristics of DFSP.

**Results:** This study systemically describes the transcriptomic characteristics of DFSP. First, we performed gene fusion analysis and identified a novel *FBN1-CSAD* fusion event in a DFSP patient with fibrosarcomatous transformation. Then, we identified *TLK2* as a biomarker for DFSP based on functional enrichment analysis, and validated its accuracy for diagnosing DFSP by immunohistochemical staining and joint analysis with public data. Finally, microenvironment analysis described the infiltration characteristics of immune and stromal cells in DFSP.

**Conclusion:** This study demonstrates that RNA-seq can serve as a promising strategy for exploring molecular mechanisms in DFSP. Our results provide new insights into accurate diagnosis and therapeutic targets of DFSP.

## Introduction

Dermatofibrosarcoma protuberans (DFSP) is a rare, low-grade cutaneous sarcoma originating from fibroblasts. According to the National Comprehensive Cancer Network (NCCN) guidelines (version 1.2020), the incidence rate of DFSP ranges from 4.2 to 4.5 cases per million people in the United States. Despite the low incidence rate, it is still regarded as the most common skin sarcoma (Ugurel et al., 2019). DFSP most commonly occurs in the trunk, followed by the extremities, head, and neck (Kreicher et al., 2016). It can be divided into dozens of variants based on the composition and microscopic characteristics, such as fibrosarcomatous (FS-DFSP), pigmented, and myxoid (Iwasaki et al., 2019). The rearrangement of chromosomes 17 and 22 leads to the fusion of the *PDGFB* (beta-type platelet-derived growth factor) gene and the *COL1A1* (collagen type 1 alpha 1) gene, which is thought to contribute to tumorigenesis. At the same time, other types of gene fusion, such as *COL6A3-PDGFD* (Dickson et al., 2018), *TNC-PDGFD* (Chen et al., 2021), and *MAP3K7CL-ERG* (Maloney et al., 2019), have been reported in some cases and contribute to the appearance of DFSP.

DFSP is usually characterized by painless, slow-growing nodules, which appear blue-purple or tan in color (Li Y. et al., 2020). However, the specific clinical manifestations of DFSP are lacking. Diagnosis of DFSP mainly depends on hematoxylin–eosin (H&E) and immunohistochemistry stains. Microscopically, tumor cells show a spindle-shaped appearance and are arranged in a storiform pattern. These spindle cells always infiltrate between fat cells and exhibit a ‘honeycomb’ appearance; however, the epidermis is usually not invaded (Kornik et al., 2012). A positive immunohistochemistry for CD34 is considered the only sensitive marker for DFSP and has been used as a diagnostic tool for decades. However, both the microscopic and immunohistochemical features mentioned above are not specific. For example, sclerotic fibroma and solitary fibrous tumors may show similar cellular arrangement features and positive immunohistochemistry for CD34 (Rapini and Golitz, 1989; Westra et al., 1994; Hanft et al., 2000). Thus, it is not easy to make an accurate and timely diagnosis.

*TLK2* (tousled-like kinases 2), a member of the tousled-like kinase (TLK) family, encodes a nuclear serine/threonine kinase. In mammals, TLKs affect genome maintenance, cell-cycle progression, and cell division, mainly by regulating histone 3 and histone 4 in S phase (Silljé et al., 1999; Silljé and Nigg, 2001; Han et al., 2005). *TLK2* is thought to be associated with the occurrence and progression of multiple diseases. Haploinsufficiency of *TLK2* may lead to a series of neurodevelopmental disorders, such as neurodevelopment delay and behavior disorders (Reijnders et al., 2018). At the same time, amplification of *TLK2* plays a crucial role in the development of cancers and is correlated with poor prognosis (Stevens et al., 2011; Kim et al., 2016; Mertins et al., 2016; Kuczler et al., 2022). According to the above observations, *TLK2* may serve as a biomarker and therapeutic target for multiple diseases.

Next-generation sequencing (NGS) has become a cost-effective technology and is widely used to survey a variety of diseases, allowing the identification of new markers for clinical diagnostics and therapeutics (Kaczkowski et al., 2016; Zhu et al., 2021). RNA sequencing (RNA-seq) allows the detection of all RNA transcripts and provides information on the transcriptional state. Currently, there are millions of RNA-seq samples stored in public databases, such as The Cancer Genome Atlas (TCGA) and Gene Expression Omnibus (GEO). However, there is an absence of RNA-seq information on DFSP in public databases. Hence, we compiled 27 samples for sequencing, including 14 DFSP samples and 13 normal adjacent tissues. Using R software, we performed gene fusion analysis and identified a novel *FBN1-CSAD* fusion event. We also screened for the differentially expressed genes (DEGs) and showed that *TLK2* served as a specific biomarker for DFSP. Finally, we compared the immune profile of the microenvironment between tumors and normal adjacent tissues, delineating an overall immune landscape of DFSP. Our study uncovered the mechanisms of DFSP at the transcriptome level and provides preliminary data that may allow accurate diagnosis and treatment in the future.

## Materials and methods

### Clinical samples

A total of 14 formalin-fixed, paraffin-embedded DFSP tissues and paired normal adjacent tissues were collected from patients at Shanghai Ninth People’s Hospital Affiliated Shanghai Jiao Tong University School of Medicine. An expert dermatopathologist confirmed all samples pathologically through hematoxylin–eosin (H&E) staining, immunohistochemistry, and fluorescence *in situ* hybridization (FISH). The study protocols were granted by the Clinical Research Ethics Committees of Shanghai Ninth People’s Hospital Affiliated Shanghai Jiao Tong University School of Medicine (Approval #: 2017-451-T3347). All the patients signed written informed consent to use their tissue samples.

### RNA sequencing and quality control

After standard RNA extraction, quality control was done using an Agilent 2100 Bioanalyzer and a NanoDrop Spectrophotometer. In the 14 paired samples, the normal adjacent tissue of Case 11 was excluded because of its low RNA concentration, and 27 samples were sequenced in total. Clean data were obtained using fastp software (version 0.20.0) and aligned to the reference genome hs37d5 by STAR alignment software (version 2.7.6a). Samples meeting the following criteria were regarded as valid data: 1) more than ∼60% reads uniquely mapped; 2) less than ∼25% reads multiply mapped or unmapped; 3) rRNA rate less than ∼15%. All the expression data were standardized and transformed as fragments per kilobase million (FPKM) and transcripts per kilobase million (TPM) for subsequent analysis.

### Gene fusion detection

Based on the results of the STAR alignment, we used Arriba (https://github.com/suhrig/arriba/) to detect fusion genes in the RNA-seq data. We defined a positive fusion event as appearing when >20 supporting reads could be detected in each sample. The tumor sample of Case 3 was omitted from the analysis because of quality issues.

### Identifying differentially expressed genes

Principal component analysis (PCA) of the top 5,000 expressed genes was conducted and visualized using the R package ggplot2 to cluster samples with similar gene expression profiles. Differentially expressed genes between tumor and normal adjacent tissue samples were assessed using the R package DESeq2 (version 1.26.0). To make the result more reliable, we only retained genes whose expression could be detected in all samples. We identified the genes which satisfied the following conditions simultaneously as significantly differentially expressed genes: 1) the adjusted *p*-value (Padj.) was less than 0.05; 2) the basemean was greater than 100.

### Functional enrichment analysis

Enrichment analysis allows us to explore gene clusters with similar function or expression trends. We performed weighted gene co-expression network analysis (WGCNA) with the R package WGCNA (version 1.70-3) for all differentially expressed genes. A soft-threshold power of 18 and module size greater than 30 genes were selected to construct a scale-free network. We merged the modules with a similarity greater than 0.75, which were considered to perform similar functions. For genes in selected modules, Gene Ontology (GO) and Kyoto Encyclopedia of Genes and Genomes (KEGG) pathway enrichment were done using the R package clusterProfiler (version 3.14.0). Significant functions and pathways were selected with Padj. < 0.05.

### Public data collection and processing

RNA-seq data of the TCGA sarcoma cohort were downloaded from the UCSC Xena database (https://xena.ucsc.edu) and selected by R (version 3.6.3). We selected soft-tissue sarcomas that occurred in the head and neck, trunk, and extremities for subsequent analysis. RUVg function in RUVSeq (version 1.27.0) was used to remove batch effects (parameter *k* = 1) between TCGA and our RNA-seq data. The receiver operating characteristic (ROC) curves and area under ROC curves (AUC) were plotted and calculated to predict the diagnostic ability of target genes.

### Immunohistochemical staining

To immunostain FAM118B and TLK2 protein in DFSP samples, formalin-fixed and paraffin-embedded specimens were cut into 4 µm thick sections and stained with standard protocols. *TLK2* polyclonal antibody (1: 100, ab224729, abcam) and *FAM118B* polyclonal antibody (1:100, PA5-59660, Thermo Fisher Scientific) were used as primary antibodies. Sections were also stained with hematoxylin and eosin for histopathological confirmation of DFSP tissue.

### Immune landscape analysis

The abundance of the tumor microenvironment was evaluated by the R package immunedeconv (version 2.0.0) and ssGSEA algorithm. The score of each immune cell type was calculated according to their characteristic gene expression. The immune score, stroma score, and microenvironment score of each sample were evaluated based on xCell algorithms.

### Statistical analysis

We used the Wilcoxon rank-sum test for paired testing of the immune landscape analysis between DFSP and normal adjacent tissues. A two-tailed *p*-value < 0.05 was considered statistically significant. All analyses were performed and visualized using R software (version 3.6.3).

## Results

### Clinical samples for RNA sequencing

**FIGURE 1:**
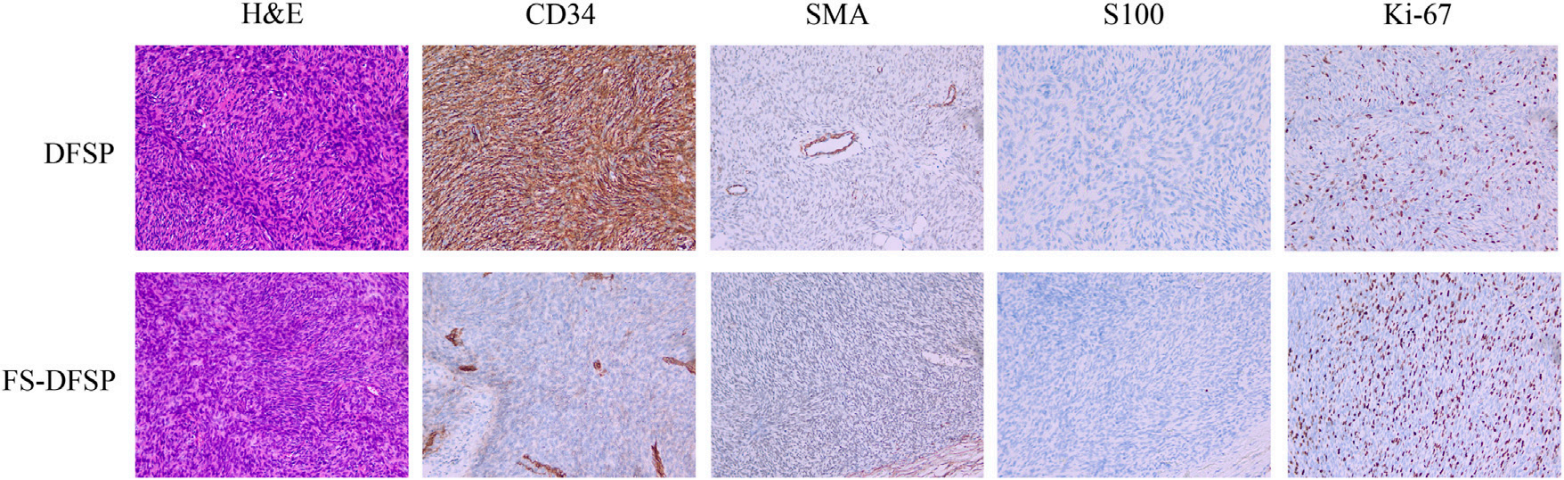
Hematoxylin–eosin (H&E) staining and immunohistochemical expression of dermatofibrosarcoma protuberans (DFSP) and dermatofibrosarcoma protuberans of fibrosarcomatous variant (FS-DFSP).

Fourteen formalin-fixed, paraffin-embedded DFSP tissues and paired normal adjacent tissues were retrospectively collected from 14 DFSP patients for RNA-seq analyses. Clinical information on the selected patients is summarized in Table 1. Of the 14 patients, nine were diagnosed with typical DFSP and five with FS-DFSP. Figure 1 shows the histological and immunohistochemical features of typical DFSP and FS-DFSP samples. FISH confirmed that 13 of the 14 patients were positive for the *COL1A1-PDGFB* fusion. No *COL1A1-PDGFB* fusion was detected in Case 10. Supplementary Figure S1 shows the histological and immunohistochemical features of the DFSP lesion of Case 10. Normal adjacent tissues were obtained from the negative margins of Mohs micrographic surgery. After quality control, we excluded the normal adjacent tissue of Case 11 owing to its low RNA concentration. Thus, RNA-seq was performed on 27 samples (14 tumors and 13 normal adjacent tissues). The alignment efficiency is presented in Supplementary Table S1. According to the criteria described in the Materials and Methods, 20 samples (13 tumors and 7 normal adjacent tissues) were included in the following analysis.

**TABLE 1:** Clinical features of 14 DFSP patients.

Case No.	Gender	Age	Tumor site	Histological variant	Primary/Recurrence	Ki-67	COL1A1-PDGFB fusion
1	F	30	Thighs	Classic	Primary	<5%	+
2	F	26	Abdomen	Classic	Primary	>20%	+
3	M	48	Chest	Classic	Primary	<5%	+
4	M	56	Abdomen	Classic	Primary	<5%	+
5	F	29	Abdomen	Classic	Primary	>20%	+
6	F	39	Shoulder	Classic	Primary	>10%	+
7	M	41	Chest	Classic	Primary	>5%	+
8	M	42	Thighs	Classic	Primary	<5%	+
9	M	12	Foot	Classic	Primary	<5%	+
10	F	55	Abdomen	Fibrosarcomatous	Recurrence	>10%	-
11	M	72	Back	Fibrosarcomatous	Recurrence	>20%	+
12	F	39	Abdomen	Fibrosarcomatous	Recurrence	>5%	+
13	F	41	Abdomen	Fibrosarcomatous	Recurrence	>5%	+
14	M	38	Abdomen	Fibrosarcomatous	Recurrence	>10%	+

### Detection of fusion genes in DFSP lesions

Gene fusions are characteristic of many sarcoma subtypes, including DFSP (Steele et al., 2019). Thus, we focused on fusion gene analysis using Arriba. Consistent with our FISH results, the *COL1A1-PDGFB* fusion gene was detected in all samples except Case 10 (a DFSP patient with fibrosarcomatous transformation). For patients with the *COL1A1-PDGFB* fusion gene, the *PDGFB* breakpoints were all located on chr 22:39631879, while the *COL1A1* breakpoints varied from chr 17:48263678 to 17:48276587. We found that case 10 harbored an *FBN1-CSAD* fusion, which has not been reported previously. The *FBN1* breakpoint was located on chr 15:48755279 and the *CSAD* breakpoint was located on chr 12:5357337. The histological and immunohistochemical features of this lesion are shown in Supplementary Figure S1. Table 2 shows the specific gene fusion profile and breakpoint location for each sample.

**TABLE 2:** The gene fusion profiles of DFSP samples.

Case no.	Fusion type	Gene1	Breakpoint	Exon	Supporting reads counts	Gene2	Breakpoint	Site	Supporting reads counts	Fusion transcript
1	Translocation	COL1A1	17:48264845	1–46	65	PDGFB	22:39631879	2–7	55	…TGG​TCC​CCG​A|GGG​GAC​CCC​A…
2	Translocation	COL1A1	17:48263678	1–49	84	PDGFB	22:39631879	2–7	144	…TGG​ATT​CCA​G|GGG​GAC​CCC​A…
4	Translocation	COL1A1	17:48271304	1–25	23	PDGFB	22:39631879	2–7	39	…AGG​TGC​TGC​T|GGG​GAC​CCC​A…
5	Translocation	COL1A1	17:48276587	1–5	155	PDGFB	22:39631879	2–7	150	… CCT​CGG​AGG​A|GGG​GAC​CCC​A…
6	Translocation	COL1A1	17:48274371	1–11	23	PDGFB	22:39631879	2–7	25	…GGG​ACA​CAG​A|GGG​GAC​CCC​A…
7	Translocation	COL1A1	17:48264845	1–46	232	PDGFB	22:39631879	2–7	204	…TGG​TCC​CCG​A|GGG​GAC​CCC​A…
8	Translocation	COL1A1	17:48264845	1–46	301	PDGFB	22:39631879	2–7	301	…TGG​TCC​CCG​A|GGG​GAC​CCC​A…
9	Translocation	COL1A1	17:48264845	1–46	299	PDGFB	22:39631879	2–7	300	…TGG​TCC​CCG​A|GGG​GAC​CCC​A…
10	Translocation	COL1A1	17:48269836	1–29	256	PDGFB	22:39631879	2–7	232	…TGG​TGA​ACA​G|GGG​GAC​CCC​A…
11	Translocation	FBN1	15:48755279	1–42	39	CSAD	12:53573378	1–2	40	…CCA​AGT​ACA​G|AAT​GAT​CCT​A…
12	Translocation	COL1A1	17:48265237	1–45	207	PDGFB	22:39631879	2–7	291	…TGG​CCC​TCC​T|GGG​GAC​CCC​A…
13	Translocation	COL1A1	17:48273675	1–14	36	PDGFB	22:39631879	2–7	40	…TGG​CCC​TGC​T|GGG​GAC​CCC​A…
14	Translocation	COL1A1	17:48265891	1–43	140	PDGFB	22:39631879	2–7	154	…TGG​TGA​GAC​T|GGG​GAC​CCC​A…

### Identification of differentially expressed genes

**FIGURE 2:**
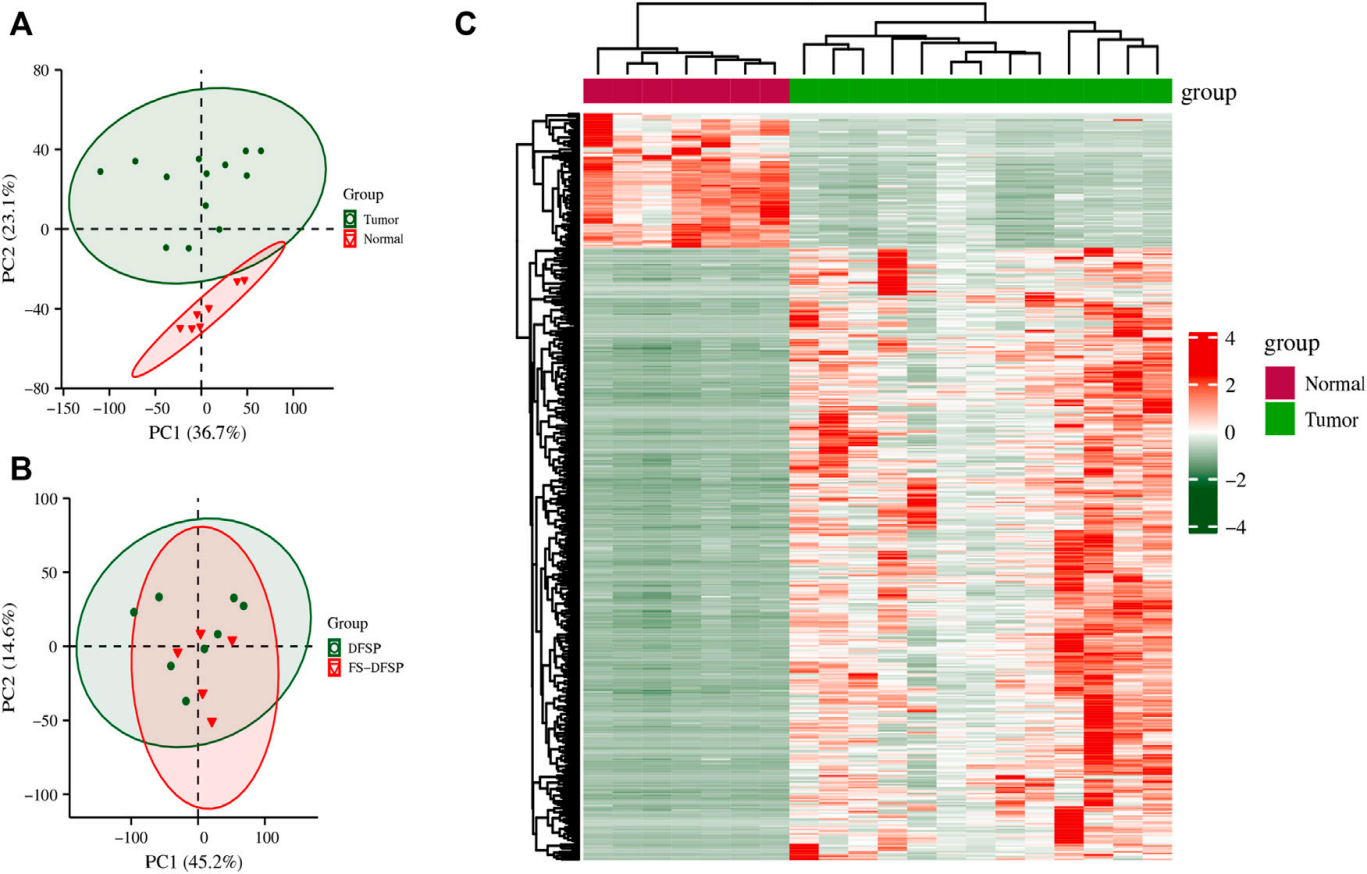
Differentially expressed genes. **(A)** Principal Component Analysis (PCA) of genes between the tumor and normal adjacent tissue groups. **(B)** PCA of genes between the dermatofibrosarcoma protuberans (DFSP) group and dermatofibrosarcoma protuberans of fibrosarcomatous variant (FS-DFSP) group. **(C)** The top 500 differentially expressed genes between the tumor and normal adjacent tissue groups. Red, upregulated; green, downregulated.

PCA maps of the top 5,000 expressed genes suggested that the gene profiles differed between the DFSP and normal adjacent tissues (Figure 2A). However, we found that the gene profiles were partially overlapping between the cluster of DFSP and FS-DFSP groups (Figure 2B). Compared with genes in normal adjacent tissues, a total of 7,540 DEGs, including 3523 upregulated and 4,017 downregulated genes, were identified in DFSP samples using the screening conditions described in the Materials and methods. The Supplementary Table S2 and Supplementary Table S3 shows the top 50 downregulated genes and up regulated genes respectively according to adjust *p*-value. The heatmap of the top 500 most differentially expressed genes is shown in Figure 2C.

### Construction of WGCNA network and identification of significant modules

**FIGURE 3:**
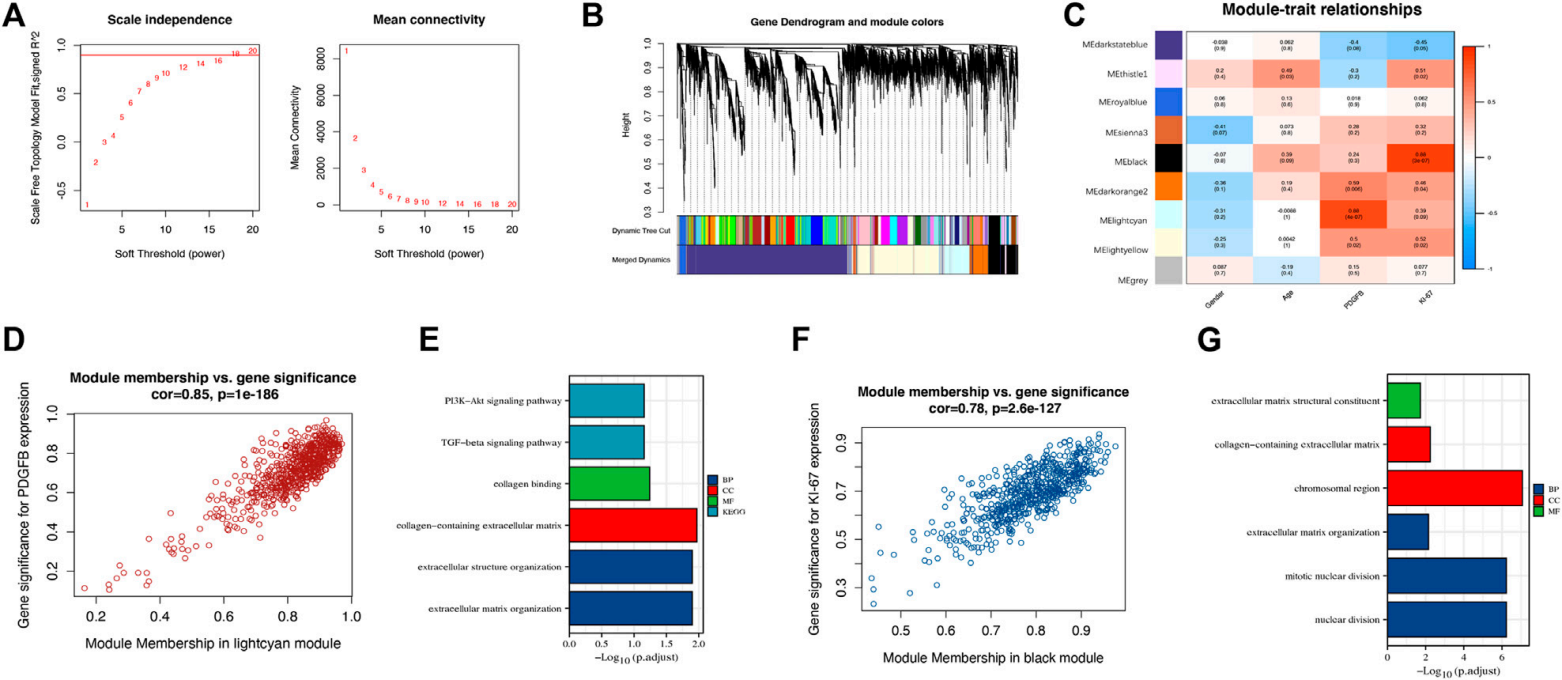
Identification of hub genes associated with tumorigenesis. **(A)** A soft-thresholding power of 18 was selected to construct a scale-free network. **(B)** Cluster dendrogram of differentially expressed genes. Each branch stands for a single gene, and each color module stands for a gene cluster performing the same function. **(C)** Correlation heatmap between gene cluster and gender, age, PDGFB, and Ki-67 expression. **(D)** The scatter plot of gene significance versus module membership in the lightcyan module. **(E)** Enrichment analysis of the function of genes in the lightcyan module. **(F)** The scatter plot of gene significance versus module membership in the black module. **(G)** Enrichment analysis of the function of genes in the black module.

We performed WGCNA on all 7,540 DEGs to explore the correlation between gene expression and clinical traits. A soft-thresholding power of 18 made the scale-free fitting index *R* ^2^ = 0.91, which led to a scale-free network (Figure 3A). Enrichment with a minimum module size of 30 and merging modules with more than 75% similarity yielded eight modules (Figure 3B). Then, each module was associated with the clinical traits (Figure 3C). We found 665 genes in the lightcyan module that correlated to the *PDGFB* expression with a correlation value of 0.88 (*p* = 4 × 10^–7^), which may be associated with DFSP tumorigenesis (Figure 3D). GO/KEGG enrichment analysis for the lightcyan module showed that genes involved in collagen and extracellular organization were enriched (Figure 3E). We also observed a strong positive correlation (*r* = 0.88, *p* = 3 × 10^–7^) between the 617 genes in the black module and Ki-67 expression, which may be associated with DFSP growth and proliferation (Figure 3F). GO/KEGG enrichment analysis for the black module showed that genes involved in cell division were enriched (Figure 3G).

Ultimately, we excluded the darkstateblue, royalblue, thistle1, and sienna3 modules from further analysis. We obtained the hub genes for the remaining modules, including *ZNF286A, FAM20C, TLK2,* and *FAM118B* (Table 3).

**TABLE 3:** Hub genes for selected modules.

Modules	Hub gene	Gene name	Gene function	LogFC	Padj
Black	ZNF286A	Zinc finger protein 286A	Remains unknow, predicted to be involved in transcriptional regulation of RNA polymerase II	1.260	<0.001
Darkorange2	FAM20C	Family with sequence similarity 20 member C	Encodes a secreted protein kinase, which involved in phosphorylates proteins associate with bone mineralization	1.730	<0.001
Lightcyan	TLK2	Tousled like kinase 2	Encodes a nuclear serine/threonine kinase which influence chromatin assembly by regulating the level of histones 3 and 4 chaperone	1.196	<0.001
Lightyellow	FAM118B	Family with sequence similarity 118 member B	Remains unknow, predicted to be participated in Cajal body organization	1.171	<0.001

### The diagnostic value of four specifically expressed hub genes in DFSP samples

**FIGURE 4:**
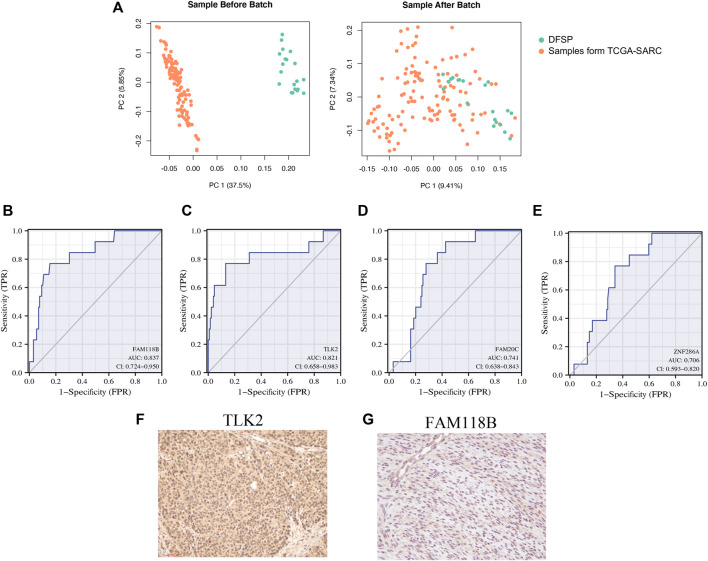
ROC curve of four specifically expressed hub genes. **(A)** Principal Component Analysis (PCA) of genes between public RNA-seq data and DFSP RNA-seq data. Orange, public RNA-seq data; green, our RNA-seq data. **(B–E)** Receiver operating characteristic (ROC) curves of **(B)** *FAM118B*; **(C)** *TLK2*; **(D)** *FAM20C*; and **(E)** *ZNF286A*. **(F,G)** Immunohistochemical staining of **(F)** *TLK2* and **(G)** *FAM118B.*

The ROC curve and AUC were used to evaluate the effectiveness of the diagnostic test. A heterogeneous group of patients with superficial tumors from the TCGA database was also enrolled for subsequent analysis to ensure both diagnostic sensitivity and specificity of selected genes. In total, 122 samples were enrolled, including undifferentiated pleomorphic sarcoma (*n* = 45), leiomyosarcoma (*n* = 29), myxofibrosarcoma (*n* = 24), malignant peripheral nerve sheath tumors (*n* = 9), dedifferentiated liposarcoma (*n* = 8), and synovial sarcoma (*n* = 7). Table 4 shows the clinical traits of selected samples in the TCGA database. After removing the batch effect (Figure 4A), the diagnostic values of *FAM118B* (AUC: 0.837; Figure 4B) and *TLK2* (AUC: 0.821; Figure 4C) were greater than 0.8. The diagnostic values of *FAM20C* (AUC: 0.741; Figure 4D) and *ZNF286A* (AUC: 0.706; Figure 4E) were weaker. By immunohistochemistry, TLK2 expression was positive while FAM118B was negative in DFSP tissues (Figure 4F). Thus, we hypothesized that *TLK2* might be a biomarker for diagnosing DFSP based on our present samples.

**TABLE 4:** Demographic and clinical characteristics of selected patients in TCGA database.

		Undifferentiated pleomorphic sarcoma	Leiomyosarcoma	Myxofibrosarcoma	Malignant peripheral Nerve sheath tumors	Dedifferentiated liposarcoma	Synovial sarcoma
Cumulative cases (n)		45	29	24	9	8	7
Age (years, mean ± SD)		68.11 ± 12.33	63.17 ± 13.84	63.75 ± 15.34	41.89 ± 16.89	63.50 ± 18.94	33.43 ± 17.61
gender (n)	Male (%)	19 (42.22%)	15 (52.72%)	10 (41.67%)	4 (44.44%)	6 (75.00%)	3 (42.86%)
	Female (%)	26 (57.78%)	14 (48.28%)	14 (58.33%)	5 (55.56%)	2 (25.00%)	4 (57.14%)
Tumor site	Trunk (%)	10 (22.22%)	8 (27.59%)	6 (25.00%)	4 (44.44%)	4 (50.00%)	1 (14.29%)
	Extremity (%)	34 (75.56%)	20 (68.97%)	18 (75.00%)	4 (44.44%)	4 (50.00%)	5 (71.43%)
	Head and neck (%)	1 (2.22%)	1 (3.44%)	0 (0.00%)	1 (11.11%)	0 (0.00%)	1 (14.29%)

### Immune microenvironment analysis of DFSP

**FIGURE 5:**
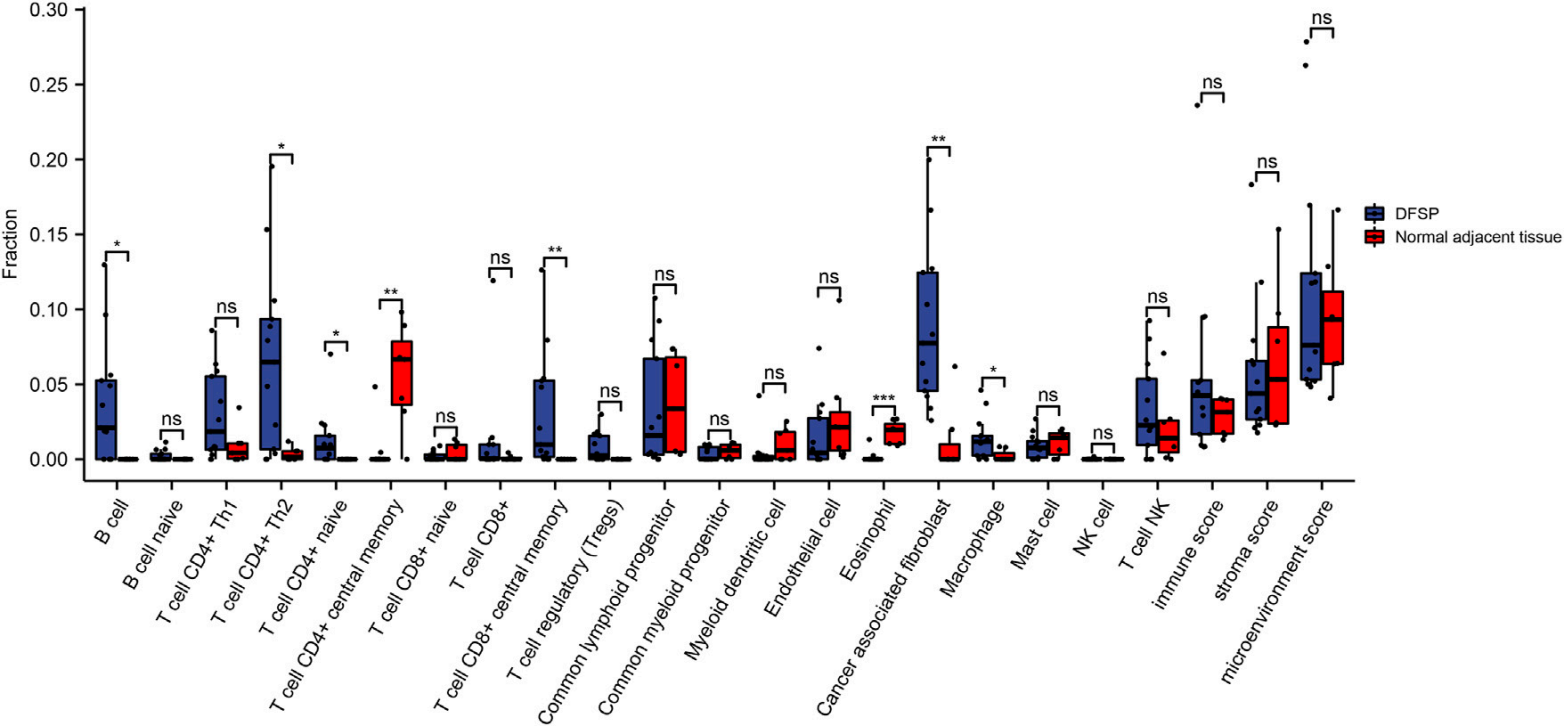
Comparison of microenvironment-related cell proportions in DFSP and normal adjacent tissue qualified by xCell algorithm. *p < 0.05, **p < 0.01.

We performed microenvironment analysis to assess the immune and stromal cell abundance in DFSP samples compared with normal tissue (Figure 5). The result of the ssGSEA algorithm showed that the expression of B cells, CD4^+^ Th2 cells, CD4^+^ naïve T cells, CD8^+^ central memory T cells, cancer-associated fibroblasts, and macrophages were significantly higher. CD4^+^ central memory T cells and eosinophils were expressed significantly lower in tumor cells. However, the immune, stroma, and microenvironment scores showed no statistical differences between the tumor and normal adjacent tissues, indicating that DFSP is an immune-cold tumor.

## Discussion

DFSP is a rare, aggressive, malignant skin tumor that accounts for about 1% of all sarcoma (Molina et al., 2018). Histologically, the tumor cells exhibit a single spindle pattern with obese or elongated wave-like nuclei, which arranged in a swirling or storiform pattern. Usually, the tumor cells infiltrate diffusely in the dermis and subcutaneous tissue, developing a typical honeycomb appearance, while the epidermis is usually unaffected. Vascular hyperplasia, granulocytic formation, nuclear fenestration and Verocay vesicle can also be found in some cases. Clinically, diagnosis is mainly dependent on morphological characteristics and positive staining for CD34. However, these characteristics significantly overlap with other soft-tissue tumors and lack specificity (Wang et al., 2011). Over decades, several immunohistochemical markers have been implicated for the accurate diagnosis of DFSP in multiple contexts, such as Apo D (Lisovsky et al., 2008), Cthrc1 (Wang et al., 2011), nestin (Sellheyer et al., 2009), the matrix metalloproteinase (MMP) family (Weinrach et al., 2004; Kim et al., 2007; Chen et al., 2012), and WT-1 (Piombino et al., 2021). However, most of these studies have focused on differential diagnosis between dermatofibroma and DFSP, and few markers have been successfully identified.

As sequencing technology develops, NGS has become an effective tool to determine disease mechanisms. Some public data repositories, such as TCGA and GEO, provide sequences and transcript information of millions of samples. In 2003, Linn et al. applied array-based comparative genomic hybridization to nine DFSP samples and identified some highly expressed genes (Linn et al., 2003). In 2020, Köster et al. used NGS to assess the spectrum of DFSP-associated mutations at the chromosome and nucleotide levels (Köster et al., 2020). Unfortunately, we did not find RNA-seq data for DFSP in public databases. Thus, we first carried out RNA-seq on 14 DFSP samples and paired normal adjacent tissues. After quality control, we systematically analyzed the transcriptomic features of DFSP.

The translocation of chromosomes 17 and 22 that generates the *COL1A1-PDGFB* fusion gene is another characteristic of DFSP. After analyzing the DFSP *COL1A1-PDGFB* fusion pattern, we found that the *PDGFB* fusion site was relatively stable (exon 2–7), while the fusion site of the *COL1A1* breakpoint varied. At the same time, other rare chromosomal translocations and gene fusions have also been found. The *COL6A3-PDGFD* fusion gene has been identified in DFSP, mostly associated with DFSP of the breast (Dadone-Montaudié et al., 2018; Dickson et al., 2018). In addition, a novel fusion event between *MAP3K7CL* and *ERG* was identified in FS-DFSP (Maloney et al., 2019). Our study included a patient who did not show the *COL1A1-PDGFB* gene fusion event. We identified a novel *FBN1-CSAD* fusion event in this patient. Fibrillin 1 (*FBN1*) is a member of the fibrillin family, which plays a crucial role in extracellular matrix assembly (Chen et al., 2018). *FBN1* expression is strongly associated with desmoplasia in ovarian cancer (Millstein et al., 2020). Cysteine sulfinic acid decarboxylase (*CSAD*) encodes a member of the decarboxylase family and plays a crucial role in cysteine metabolism. However, to the best of our knowledge, no research has shown that *CSAD* correlates with tumor progression. As the fibrosarcomatous transformation of DFSP is complex, identification of this novel *FBN1-CSAD* fusion gene is vital in delving into the potential molecular mechanisms of DFSP and its fibrosarcomatous transformation.

Through DEG and WGCNA analyses, we identified four potential biomarkers for DFSP. *FAM118B* is thought to correlate with the composition and function of Cajal bodies, compartments for the biogenesis of small nuclear ribonucleoproteins (Li et al., 2014). Research found that *FAM118B* promotes NF2 wild-type meningiomas through the formation of *YAP-FAM118B* fusion genes (Szulzewsky et al., 2020; Schieffer et al., 2021). As a member of the zinc finger protein family, *ZNF286A* is predicted to regulate the activity of DNA-binding transcription factors. Although other members of this family, such as *ZNF224* (Cesaro et al., 2021) and *ZNF217* (Li et al., 2021), play important roles in cancer development, the role of *ZNF286A* has not been studied in any disease. *FAM20C* is a secreted protein with atypical kinase activity, which plays important roles in biomineralization, cell migration, and wound healing (Xu et al., 2021). Studies have indicated that *FAM20C* might be a biomarker for diverse cancers, including triple-negative breast cancer (Tagliabracci et al., 2015), lung adenocarcinoma (Li H. et al., 2020), and stomach adenocarcinoma (Liu et al., 2021). *TLK2*, a nuclear serine-threonine kinase, binds at the H3 binding site to promote *ASF1* (histone chaperone protein anti-silencing factor 1) phosphorylation and cell growth (Simon et al., 2022). *TLK2* amplifies and serves as an oncogene in multiple diseases (Kim et al., 2016; Lelieveld et al., 2016; Kuczler et al., 2022). *TLK2* depletion activates the cGAS–STRING–TBK1 innate immune axis, and a high *TLK2* level contributes to immune evasion of cancers positive for alternative lengthening of telomeres (Segura-Bayona et al., 2020). The mRNA expression levels of these four genes were increased in DFSP samples and were closely related to tumorigenesis through WGCNA and GO/KEGG enrichment analysis. Subsequently, a joint analysis with the other six types of soft-tissue sarcoma samples was performed to verify their diagnostic efficacy. The ROC curves suggested that *FAM20C* and *ZNF286A* have a lower specificity for differentiating DFSP from other tumors (AUC <0.75) compared with *FAM118B* and *TLK2* (AUC >0.8). Immunohistochemical staining revealed the presence of *TLK2* in DFSP cells while the expression of *FAM118B* was absent. Therefore, we believe that *TLK2* can serve as a biomarker in DFSP.

Furthermore, the microenvironment landscapes showed that the infiltration of non-tumor cells differed between tumor and normal tissues. In general, in most cancers, high infiltration of CD8^+^ T cells, Th1 cells, and NK cells are indicative of good prognosis, while the infiltration of macrophages, Th17 cells, and Th2 cells are associated with bad prognosis (Bruni et al., 2020). We found increased infiltration of Th2 cells and macrophages (indicating bad prognosis) and no significant difference in CD8^+^ T cells, Th1 cells, or NK cells (indicating good prognosis), which partially explains the local invasion properties of DFSP. Meanwhile, it is worth noting that cancer-associated fibroblasts were significantly upregulated in DFSP. Cancer-associated fibroblasts are a heterogeneous population of stromal cells that play a vital role in tumor progression and drug resistance (Czekay et al., 2022). They also show intrastromal crosstalk with other types of immune cells, including T cells, dendritic cells, and macrophages (Saw et al., 2022). Targeting cancer-associated fibroblasts directly or indirectly can inhibit tumor malignancy in solid tumors (Saw et al., 2022). Targeted interventions of cancer-associated fibroblasts could serve as a promising measure to inhibit invasion of DFSP.

In conclusion, this study comprehensively describes the transcriptomic characteristics of DFSP and identifies a new *FBN1-CSAD* fusion gene. At the same time, we have identified *TLK2* as a potential biomarker for diagnosis and treatment. Moreover, we have also provided new insight into microenvironment infiltration in DFSP that may identify potential targets for immunotherapy. However, there are several limitations of this study. First, owing to the rarity of this disease, the RNA-seq sample size was limited. Second, validating the diagnostic efficacy of *TLK2* in *in vivo* experiments would be advantageous. In addition, the influence of the tumor microenvironment in DFSP has not been fully clarified and requires further exploration.

## Data Availability

All datasets generated for this study are included in the article/supplementary material.

## Supplementary material

Supplementary Figure S1 Histological and immunohistochemical features of the lesion in Case 10. The hematoxylin–eosin (H&E) staining shows heterogeneity and irregular arrangement of the spindle tumor cells. The immunohistochemistry shows that the tumor is positive for CD34 and negative for SMA and S100. The positive rate for Ki-67 is approximately 10%.

## References

[B1] Bruni D. Angell H. K. Galon J. (2020). The immune contexture and Immunoscore in cancer prognosis and therapeutic efficacy. Nat. Rev. Cancer 20, 662–680. 10.1038/s41568-020-0285-7 32753728

[B2] Cesaro E. Lupo A. Rapuano R. Pastore A. Grosso M. Costanzo P. (2021). ZNF224 protein: Multifaceted functions based on its molecular partners. Molecules 26, 6296. 10.3390/molecules26206296 34684876 PMC8537547

[B3] Chen M. Yao B. Yang Q. Deng J. Song Y. Sui T. (2018). Truncated C-terminus of fibrillin-1 induces Marfanoid-progeroid-lipodystrophy (MPL) syndrome in rabbit. Dis. Model. Mech. 11, dmm031542. 10.1242/dmm.031542 29666143 PMC5963856

[B4] Chen Y.-T. Chen W.-T. Huang W.-T. Wu C.-C. Chai C.-Y. (2012). Expression of MMP-2, MMP-9 and MMP-11 in dermatofibroma and dermatofibrosarcoma protuberans. Kaohsiung J. Med. Sci. 28, 545–549. 10.1016/j.kjms.2012.04.017 23089320 PMC11915974

[B5] Chen Y. Shi Y.-Z. Feng X.-H. Wang X.-T. He X.-L. Zhao M. (2021). Novel TNC-PDGFD fusion in fibrosarcomatous dermatofibrosarcoma protuberans: A case report. Diagn. Pathol. 16, 63. 10.1186/s13000-021-01123-1 34256767 PMC8276425

[B6] Czekay R.-P. Cheon D.-J. Samarakoon R. Kutz S. M. Higgins P. J. (2022). Cancer-associated fibroblasts: Mechanisms of tumor progression and novel therapeutic targets. Cancers (Basel) 14, 1231. 10.3390/cancers14051231 35267539 PMC8909913

[B7] Dadone-Montaudié B. Alberti L. Duc A. Delespaul L. Lesluyes T. Pérot G. (2018). Alternative PDGFD rearrangements in dermatofibrosarcomas protuberans without PDGFB fusions. Mod. Pathol. 31, 1683–1693. 10.1038/s41379-018-0089-4 29955147

[B8] Dickson B. C. Hornick J. L. Fletcher C. D. M. Demicco E. G. Howarth D. J. Swanson D. (2018). Dermatofibrosarcoma protuberans with a novel COL6A3-PDGFD fusion gene and apparent predilection for breast. Genes. Chromosom. Cancer 57, 437–445. 10.1002/gcc.22663 30014607 PMC6762016

[B9] Han Z. Riefler G. M. Saam J. R. Mango S. E. Schumacher J. M. (2005). The *C. elegans* Tousled-like kinase contributes to chromosome segregation as a substrate and regulator of the Aurora B kinase. Curr. Biol. 15, 894–904. 10.1016/j.cub.2005.04.019 15916946 PMC2653428

[B10] Hanft V. N. Shea C. R. McNutt N. S. Pullitzer D. Horenstein M. G. Prieto V. G. (2000). Expression of CD34 in sclerotic (“plywood”) fibromas. Am. J. Dermatopathol. 22, 17–21. 10.1097/00000372-200002000-00003 10698210

[B11] Iwasaki T. Yamamoto H. Oda Y. (2019). Current update on the molecular biology of cutaneous sarcoma: Dermatofibrosarcoma protuberans. Curr. Treat. Options Oncol. 20, 29. 10.1007/s11864-019-0628-3 30874910

[B12] Kaczkowski B. Tanaka Y. Kawaji H. Sandelin A. Andersson R. Itoh M. (2016). Transcriptome analysis of recurrently deregulated genes across multiple cancers identifies new pan-cancer biomarkers. Cancer Res. 76, 216–226. 10.1158/0008-5472.CAN-15-0484 26552699

[B13] Kim H. J. Lee J. Y. Kim S. H. Seo Y. J. Lee J. H. Park J. K. (2007). Stromelysin-3 expression in the differential diagnosis of dermatofibroma and dermatofibrosarcoma protuberans: Comparison with factor XIIIa and CD34. Br. J. Dermatol. 157, 319–324. 10.1111/j.1365-2133.2007.08033.x 17596171

[B14] Kim J.-A. Tan Y. Wang X. Cao X. Veeraraghavan J. Liang Y. (2016). Comprehensive functional analysis of the tousled-like kinase 2 frequently amplified in aggressive luminal breast cancers. Nat. Commun. 7, 12991. 10.1038/ncomms12991 27694828 PMC5064015

[B15] Kornik R. I. Muchard L. K. Teng J. M. (2012). Dermatofibrosarcoma protuberans in children: An update on the diagnosis and treatment. Pediatr. Dermatol. 29, 707–713. 10.1111/j.1525-1470.2012.01767.x 22780227

[B16] Köster J. Arbajian E. Viklund B. Isaksson A. Hofvander J. Haglund F. (2020). Genomic and transcriptomic features of dermatofibrosarcoma protuberans: Unusual chromosomal origin of the COL1A1-PDGFB fusion gene and synergistic effects of amplified regions in tumor development. Cancer Genet. 241, 34–41. 10.1016/j.cancergen.2019.12.001 31870844

[B17] Kreicher K. L. Kurlander D. E. Gittleman H. R. Barnholtz-Sloan J. S. Bordeaux J. S. (2016). Incidence and survival of primary dermatofibrosarcoma protuberans in the United States. Dermatol. Surg. 42 Suppl 1, S24–S31. 10.1097/DSS.0000000000000300 26730971

[B18] Kuczler M. D. Zieren R. C. Dong L. de Reijke T. M. Pienta K. J. Amend S. R. (2022). Advancements in the identification of EV derived mRNA biomarkers for liquid biopsy of clear cell renal cell carcinomas. Urology 160, 87–93. 10.1016/j.urology.2021.11.002 34793840 PMC8882144

[B19] Lelieveld S. H. Reijnders M. R. F. Pfundt R. Yntema H. G. Kamsteeg E.-J. de Vries P. (2016). Meta-analysis of 2, 104 trios provides support for 10 new genes for intellectual disability. Nat. Neurosci. 19, 1194–1196. 10.1038/nn.4352 27479843

[B20] Li H. Tong L. Tao H. Liu Z. (2020a). Genome-wide analysis of the hypoxia-related DNA methylation-driven genes in lung adenocarcinoma progression. Biosci. Rep. 40, BSR20194200. 10.1042/BSR20194200 32031203 PMC7033312

[B21] Li Y. Fong K.-W. Tang M. Han X. Gong Z. Ma W. (2014). Fam118B, a newly identified component of Cajal bodies, is required for Cajal body formation, snRNP biogenesis and cell viability. J. Cell. Sci. 127, 2029–2039. 10.1242/jcs.143453 24569877 PMC4004977

[B22] Li Y. Wang C. Yang K. Peng S. Wang Q. Chen S. (2020b). Clinical features of dermatofibrosarcoma protuberans and risk factors for local recurrence after Mohs micrographic surgery. J. Am. Acad. Dermatol. 82, 1219–1221. 10.1016/j.jaad.2019.09.034 31560980

[B23] Li Y. Wu H. Wang Q. Xu S. (2021). ZNF217: The cerberus who fails to guard the gateway to lethal malignancy. Am. J. Cancer Res. 11, 3378–3405. 34354851 PMC8332857

[B24] Linn S. C. West R. B. Pollack J. R. Zhu S. Hernandez-Boussard T. Nielsen T. O. (2003). Gene expression patterns and gene copy number changes in dermatofibrosarcoma protuberans. Am. J. Pathol. 163, 2383–2395. 10.1016/S0002-9440(10)63593-6 14633610 PMC1892373

[B25] Lisovsky M. Hoang M. P. Dresser K. A. Kapur P. Bhawan J. Mahalingam M. (2008). Apolipoprotein D in CD34-positive and CD34-negative cutaneous neoplasms: A useful marker in differentiating superficial acral fibromyxoma from dermatofibrosarcoma protuberans. Mod. Pathol. 21, 31–38. 10.1038/modpathol.3800971 17885669

[B26] Liu X. Zhan Y. Xu W. Liu X. Geng Y. Liu L. (2021). Prognostic and immunological role of Fam20C in pan-cancer. Biosci. Rep. 41, BSR20201920. 10.1042/BSR20201920 33306121 PMC7786334

[B27] Maloney N. Bridge J. A. de Abreu F. Korkolopoulou P. Sakellariou S. Linos K. (2019). A novel MAP3K7CL-ERG fusion in a molecularly confirmed case of dermatofibrosarcoma protuberans with fibrosarcomatous transformation. J. Cutan. Pathol. 46, 532–537. 10.1111/cup.13469 30950098

[B28] Mertins P. Mani D. R. Ruggles K. V. Gillette M. A. Clauser K. R. Wang P. (2016). Proteogenomics connects somatic mutations to signalling in breast cancer. Nature 534, 55–62. 10.1038/nature18003 27251275 PMC5102256

[B29] Millstein J. Budden T. Goode E. L. Anglesio M. S. Talhouk A. Intermaggio M. P. (2020). Prognostic gene expression signature for high-grade serous ovarian cancer. Ann. Oncol. 31, 1240–1250. 10.1016/j.annonc.2020.05.019 32473302 PMC7484370

[B30] Molina A. S. Duprat Neto J. P. Bertolli E. da Cunha I. W. Fregnani J. H. T. G. Figueiredo P. H. M. (2018). Relapse in dermatofibrosarcoma protuberans: A histological and molecular analysis. J. Surg. Oncol. 117, 845–850. 10.1002/jso.25039 29509956

[B31] Piombino E. Broggi G. Barbareschi M. Castorina S. Parenti R. Bartoloni G. (2021). Wilms’ tumor 1 (WT1): A novel immunomarker of dermatofibrosarcoma protuberans-an immunohistochemical study on a series of 114 cases of bland-looking mesenchymal spindle cell lesions of the dermis/subcutaneous tissues. Cancers (Basel) 13, E252. 10.3390/cancers13020252 33445443 PMC7826654

[B32] Rapini R. P. Golitz L. E. (1989). Sclerotic fibromas of the skin. J. Am. Acad. Dermatol. 20, 266–271. 10.1016/s0190-9622(89)70033-5 2464630

[B33] Reijnders M. R. F. Miller K. A. Alvi M. Goos J. A. C. Lees M. M. de Burca A. (2018). De novo and inherited loss-of-function variants in TLK2: Clinical and genotype-phenotype evaluation of a distinct neurodevelopmental disorder. Am. J. Hum. Genet. 102, 1195–1203. 10.1016/j.ajhg.2018.04.014 29861108 PMC5992133

[B34] Saw P. E. Chen J. Song E. (2022). Targeting CAFs to overcome anticancer therapeutic resistance. Trends Cancer 8, 527–555. 10.1016/j.trecan.2022.03.001 35331673

[B35] Schieffer K. M. Agarwal V. LaHaye S. Miller K. E. Koboldt D. C. Lichtenberg T. (2021). YAP1-FAM118B fusion defines a rare subset of childhood and Young adulthood meningiomas. Am. J. Surg. Pathol. 45, 329–340. 10.1097/PAS.0000000000001597 33074854

[B36] Segura-Bayona S. Villamor-Payà M. Attolini C. S.-O. Koenig L. M. Sanchiz-Calvo M. Boulton S. J. (2020). Tousled-like kinases suppress innate immune signaling triggered by alternative lengthening of telomeres. Cell. Rep. 32, 107983. 10.1016/j.celrep.2020.107983 32755577 PMC7408502

[B37] Sellheyer K. Nelson P. Krahl D. (2009). Dermatofibrosarcoma protuberans: A tumour of nestin-positive cutaneous mesenchymal stem cells? Br. J. Dermatol. 161, 1317–1322. 10.1111/j.1365-2133.2009.09363.x 19659472

[B38] Silljé H. H. Nigg E. A. (2001). Identification of human Asf1 chromatin assembly factors as substrates of Tousled-like kinases. Curr. Biol. 11, 1068–1073. 10.1016/s0960-9822(01)00298-6 11470414

[B39] Silljé H. H. Takahashi K. Tanaka K. Van Houwe G. Nigg E. A. (1999). Mammalian homologues of the plant Tousled gene code for cell-cycle-regulated kinases with maximal activities linked to ongoing DNA replication. EMBO J. 18, 5691–5702. 10.1093/emboj/18.20.5691 10523312 PMC1171636

[B40] Simon B. Lou H. J. Huet-Calderwood C. Shi G. Boggon T. J. Turk B. E. (2022). Tousled-like kinase 2 targets ASF1 histone chaperones through client mimicry. Nat. Commun. 13, 749. 10.1038/s41467-022-28427-0 35136069 PMC8826447

[B41] Steele C. D. Tarabichi M. Oukrif D. Webster A. P. Ye H. Fittall M. (2019). Undifferentiated sarcomas develop through distinct evolutionary pathways. Cancer Cell. 35, 441–456. e8. 10.1016/j.ccell.2019.02.002 30889380 PMC6428691

[B42] Stevens K. N. Wang X. Fredericksen Z. Pankratz V. S. Cerhan J. Vachon C. M. (2011). Evaluation of associations between common variation in mitotic regulatory pathways and risk of overall and high grade breast cancer. Breast Cancer Res. Treat. 129, 617–622. 10.1007/s10549-011-1587-y 21607584 PMC3508696

[B43] Szulzewsky F. Arora S. Hoellerbauer P. King C. Nathan E. Chan M. (2020). Comparison of tumor-associated YAP1 fusions identifies a recurrent set of functions critical for oncogenesis. Genes. Dev. 34, 1051–1064. 10.1101/gad.338681.120 32675324 PMC7397849

[B44] Tagliabracci V. S. Wiley S. E. Guo X. Kinch L. N. Durrant E. Wen J. (2015). A single kinase generates the majority of the secreted phosphoproteome. Cell. 161, 1619–1632. 10.1016/j.cell.2015.05.028 26091039 PMC4963185

[B45] Ugurel S. Kortmann R.-D. Mohr P. Mentzel T. Garbe C. Breuninger H. (2019). S1 guidelines for dermatofibrosarcoma protuberans (DFSP) - update 2018. J. Dtsch. Dermatol. Ges. 17, 663–668. 10.1111/ddg.13849 31115967

[B46] Wang L. Xiang Y. N. Zhang Y. H. Tu Y. T. Chen H. X. (2011). Collagen triple helix repeat containing-1 in the differential diagnosis of dermatofibrosarcoma protuberans and dermatofibroma. Br. J. Dermatol. 164, 135–140. 10.1111/j.1365-2133.2010.10050.x 20849518

[B47] Weinrach D. M. Wang K. L. Wiley E. L. Laskin W. B. (2004). Immunohistochemical expression of matrix metalloproteinases 1, 2, 9, and 14 in dermatofibrosarcoma protuberans and common fibrous histiocytoma (dermatofibroma). Arch. Pathol. Lab. Med. 128, 1136–1141. 10.1043/1543-2165(2004)128<1136:IEOMMA>2.0.CO;2 15387709

[B48] Westra W. H. Gerald W. L. Rosai J. (1994). Solitary fibrous tumor. Consistent CD34 immunoreactivity and occurrence in the orbit. Am. J. Surg. Pathol. 18, 992–998. 10.1097/00000478-199410000-00003 7522416

[B49] Xu R. Tan H. Zhang J. Yuan Z. Xie Q. Zhang L. (2021). Fam20C in human diseases: Emerging biological functions and therapeutic implications. Front. Mol. Biosci. 8, 790172. 10.3389/fmolb.2021.790172 34988120 PMC8721277

[B50] Zhu R. Yan J. Li B. Tan F. Yan W. Shen J. (2021). Determination of COL1A1-PDGFB breakpoints by next-generation sequencing in the molecular diagnosis of dermatofibrosarcoma protuberans. Exp. Mol. Pathol. 122, 104672. 10.1016/j.yexmp.2021.104672 34371012

