# Supplementary material for: Comprehensive analysis of transcriptome characteristics and identification of TLK2 as a potential biomarker in dermatofibrosarcoma protuberans
Source: Front Genet. 2022 Sep 5;13:926282. doi: 10.3389/fgene.2022.926282 (PMC9483842; doi:10.3389/fgene.2022.926282)
Supplement: Supplementary file 1 [file Table1.DOCX]

| **Supplementary Table I: Summary of orginal RNA sequencing data** | | | | | | | | | | | | |
| --- | --- | --- | --- | --- | --- | --- | --- | --- | --- | --- | --- | --- |
| SampleID | Tussue type | Total Reads | Mapped Reads | Mapped Rate(%) | Unique Mapped Reads | Unique  Mapped Rate(%) | Multiple Mapped Rate(%) | UnMapped Rate(%) | rRNA Rate(%) | Exonic Rate(%) | Intronic Rate(%) | Intergenic Rate(%) |
| 1 | tumor | 282548970 | 238551724 | 84.42 | 220420774 | 78.01 | 6.41 | 15.58 | 10.21 | 42.86 | 53.81 | 3.33 |
| 2 | tumor | 230401578 | 209192574 | 90.79 | 198442646 | 86.13 | 4.66 | 9.21 | 2.08 | 34.29 | 61.43 | 4.28 |
| 3 | tumor | 273299230 | 181744890 | 66.5 | 159804976 | 58.47 | 8.03 | 33.5 | 34.24 | 37.16 | 59.41 | 3.43 |
| 4 | tumor | 250622576 | 202682134 | 80.87 | 188200062 | 75.09 | 5.78 | 19.13 | 2.55 | 22.35 | 73.94 | 3.7 |
| 5 | tumor | 198076690 | 176955950 | 89.34 | 165965764 | 83.79 | 5.55 | 10.66 | 2.68 | 37.76 | 58.65 | 3.59 |
| 6 | tumor | 247466942 | 200288662 | 80.94 | 179279736 | 72.45 | 8.49 | 19.06 | 8.16 | 33.1 | 63.44 | 3.46 |
| 7 | tumor | 250230998 | 221459608 | 88.5 | 204311026 | 81.65 | 6.85 | 11.5 | 1.65 | 35.2 | 61.68 | 3.11 |
| 8 | tumor | 241974980 | 213032464 | 88.04 | 196933458 | 81.39 | 6.65 | 11.96 | 1.39 | 37.06 | 59.35 | 3.59 |
| 9 | tumor | 220537856 | 206222938 | 93.51 | 195640788 | 88.71 | 4.8 | 6.49 | 0.96 | 43.34 | 53.76 | 2.9 |
| 10 | tumor | 227878750 | 179090416 | 78.6 | 162971426 | 71.52 | 7.08 | 21.4 | 7.73 | 31.01 | 65.17 | 3.82 |
| 11 | tumor | 215335226 | 197741468 | 91.83 | 187642050 | 87.14 | 4.69 | 8.17 | 1.29 | 37.27 | 59.38 | 3.35 |
| 12 | tumor | 250919418 | 224615720 | 89.52 | 186531630 | 74.34 | 15.18 | 10.48 | 4.29 | 65.7 | 31.82 | 2.48 |
| 13 | tumor | 325531252 | 283162070 | 86.98 | 242790296 | 74.58 | 12.4 | 13.02 | 7.96 | 60.14 | 37.03 | 2.83 |
| 14 | tumor | 230680276 | 197762446 | 85.73 | 175576756 | 76.11 | 9.62 | 14.27 | 15.93 | 63.43 | 33.59 | 2.98 |
| 15 | normal adjacent tissue | 233379206 | 196852726 | 84.35 | 182600528 | 78.24 | 6.11 | 15.65 | 9 | 43.36 | 52.79 | 3.85 |
| 16 | normal adjacent tissue | 216297104 | 183115208 | 84.67 | 169983344 | 78.59 | 6.08 | 15.33 | 13.72 | 35.03 | 60.58 | 4.39 |
| 17 | normal adjacent tissue | 264556260 | 191754406 | 72.48 | 172699892 | 65.28 | 7.2 | 27.52 | 57.75 | 42.42 | 53.79 | 3.79 |
| 18 | normal adjacent tissue | 382265906 | 271233432 | 70.95 | 239802950 | 62.73 | 8.22 | 29.05 | 71.85 | 53.05 | 44.06 | 2.88 |
| 19 | normal adjacent tissue | 214339872 | 196168312 | 91.52 | 183475616 | 85.6 | 5.92 | 8.48 | 3.94 | 46.05 | 50.73 | 3.23 |
| 20 | normal adjacent tissue | 239598610 | 211040650 | 88.08 | 187011396 | 78.05 | 10.03 | 11.92 | 13 | 56.48 | 40.69 | 2.83 |
| 21 | normal adjacent tissue | 256155778 | 222062180 | 86.7 | 209220850 | 81.68 | 5.02 | 13.3 | 3.56 | 39.62 | 56.88 | 3.51 |
| 22 | normal adjacent tissue | 232738450 | 195732826 | 84.1 | 182276008 | 78.32 | 5.78 | 15.9 | 10.65 | 32.82 | 63.08 | 4.1 |
| 23 | normal adjacent tissue | 258690298 | 192764706 | 74.51 | 165312882 | 63.9 | 10.61 | 25.49 | 13.53 | 61.84 | 35.79 | 2.37 |
| 24 | normal adjacent tissue | 384919312 | 295079370 | 76.66 | 264023654 | 68.59 | 8.07 | 23.34 | 63.75 | 53.5 | 43.41 | 3.09 |
| 25 | normal adjacent tissue | 247727334 | 180325376 | 72.79 | 165038244 | 66.62 | 6.17 | 27.21 | 25.39 | 27.56 | 68.37 | 4.07 |
| 26 | normal adjacent tissue | 252148520 | 179964030 | 71.38 | 163111238 | 64.69 | 6.69 | 28.62 | 17.66 | 39.66 | 56.74 | 3.6 |
| 27 | normal adjacent tissue | 338043950 | 235055838 | 69.53 | 206447568 | 61.07 | 8.46 | 30.47 | 70.59 | 46.7 | 49.62 | 3.68 |
