# Supplementary figures and images for: Comprehensive analysis of transcriptome characteristics and identification of TLK2 as a potential biomarker in dermatofibrosarcoma protuberans
Source: Front Genet. 2022 Sep 5;13:926282. doi: 10.3389/fgene.2022.926282 (PMC9483842; doi:10.3389/fgene.2022.926282)

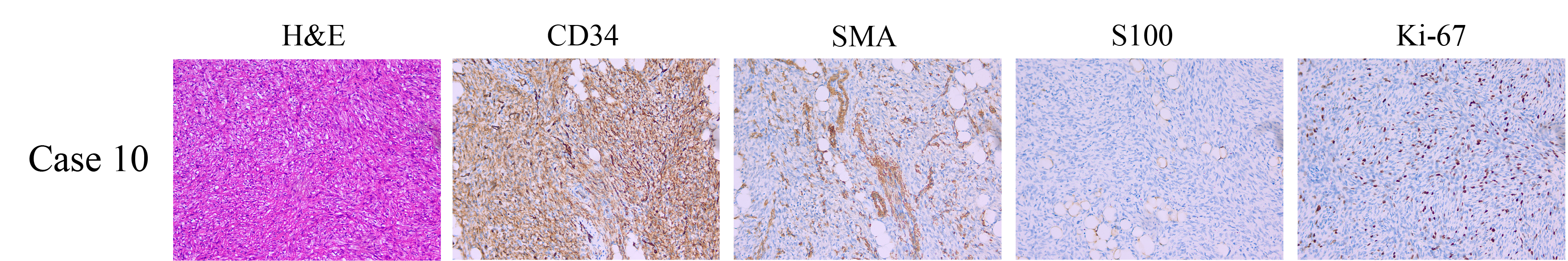

Supplement: Supplementary file 2 [file Image1.TIF]
