# Supplementary material for: Comprehensive analysis of transcriptome characteristics and identification of TLK2 as a potential biomarker in dermatofibrosarcoma protuberans
Source: Front Genet. 2022 Sep 5;13:926282. doi: 10.3389/fgene.2022.926282 (PMC9483842; doi:10.3389/fgene.2022.926282)
Supplement: Supplementary file 3 [file Table2.DOCX]

| **Supplementary Table II: Top 50 downregulate genes in DFSP** | | | |
| --- | --- | --- | --- |
| Gene name | Ensembl ID | Log2FoldChange | Padj |
| ZNF273 | ENSG00000198039 | -3.1402917 | 2.66E-22 |
| PLEKHH1 | ENSG00000054690 | -2.5728264 | 1.20E-19 |
| ATL2 | ENSG00000119787 | -1.79344 | 3.35E-19 |
| IL6 | ENSG00000136244 | -7.59193 | 1.03E-18 |
| RDH13 | ENSG00000160439 | -2.4469639 | 4.50E-17 |
| HSBP1L1 | ENSG00000226742 | -1.8979608 | 1.01E-16 |
| C1orf74 | ENSG00000162757 | -3.0677815 | 4.42E-16 |
| C11orf80 | ENSG00000173715 | -1.7164862 | 6.45E-16 |
| PHLDB3 | ENSG00000176531 | -2.2418331 | 6.99E-16 |
| SLC4A11 | ENSG00000088836 | -3.9443363 | 9.95E-16 |
| HBA1 | ENSG00000206172 | -4.9663058 | 2.04E-15 |
| HBA2 | ENSG00000188536 | -4.9586194 | 2.42E-15 |
| EDAR | ENSG00000135960 | -4.8672777 | 2.80E-15 |
| ERMP1 | ENSG00000099219 | -1.1944257 | 6.82E-15 |
| ZDHHC11 | ENSG00000188818 | -4.2354239 | 1.06E-14 |
| RFX2 | ENSG00000087903 | -2.4049103 | 1.17E-14 |
| IGSF9 | ENSG00000085552 | -3.6838412 | 1.38E-14 |
| C4orf19 | ENSG00000154274 | -2.064462 | 2.32E-14 |
| SELE | ENSG00000007908 | -4.430339 | 2.68E-14 |
| SYTL1 | ENSG00000142765 | -2.9060222 | 2.71E-14 |
| VPS13D | ENSG00000048707 | -1.3983835 | 3.03E-14 |
| KIAA1522 | ENSG00000162522 | -3.3018401 | 3.63E-14 |
| PM20D1 | ENSG00000162877 | -6.1295471 | 8.24E-14 |
| EPN2 | ENSG00000072134 | -1.2590313 | 1.08E-13 |
| AWAT2 | ENSG00000147160 | -8.2441314 | 1.60E-13 |
| CDA | ENSG00000158825 | -3.4381907 | 1.71E-13 |
| SMG1 | ENSG00000157106 | -0.8618411 | 1.82E-13 |
| C2CD4B | ENSG00000205502 | -4.0859207 | 2.12E-13 |
| DOCK3 | ENSG00000088538 | -3.3607749 | 2.61E-13 |
| SH3GLB2 | ENSG00000148341 | -1.764637 | 4.45E-13 |
| CARMIL3 | ENSG00000186648 | -2.8314519 | 6.02E-13 |
| PLXNB1 | ENSG00000164050 | -1.8977354 | 8.58E-13 |
| ITGB6 | ENSG00000115221 | -3.110682 | 9.83E-13 |
| TPPP | ENSG00000171368 | -3.3834237 | 1.02E-12 |
| HBB | ENSG00000244734 | -4.851964 | 1.46E-12 |
| ITPR2 | ENSG00000123104 | -2.5499855 | 1.53E-12 |
| PPP2R5A | ENSG00000066027 | -1.3397397 | 1.64E-12 |
| SNCA | ENSG00000145335 | -3.1064064 | 1.64E-12 |
| NFKBIZ | ENSG00000144802 | -2.2942173 | 1.78E-12 |
| RALGDS | ENSG00000160271 | -1.971612 | 2.11E-12 |
| CCDC120 | ENSG00000147144 | -1.3856319 | 2.19E-12 |
| HSD11B2 | ENSG00000176387 | -3.8863428 | 2.23E-12 |
| LONRF1 | ENSG00000154359 | -2.0602643 | 2.73E-12 |
| LMNTD2 | ENSG00000185522 | -3.1856693 | 2.90E-12 |
| COX6B2 | ENSG00000160471 | -2.9581742 | 3.81E-12 |
| FBXL16 | ENSG00000127585 | -2.197222 | 5.34E-12 |
| EML2 | ENSG00000125746 | -1.5452557 | 6.46E-12 |
| TACC2 | ENSG00000138162 | -3.0638328 | 6.61E-12 |
| CAMK2B | ENSG00000058404 | -2.4389854 | 6.76E-12 |
| DGAT2L6 | ENSG00000184210 | -5.2345754 | 6.88E-12 |
