# Supplementary material for: Comprehensive analysis of transcriptome characteristics and identification of TLK2 as a potential biomarker in dermatofibrosarcoma protuberans
Source: Front Genet. 2022 Sep 5;13:926282. doi: 10.3389/fgene.2022.926282 (PMC9483842; doi:10.3389/fgene.2022.926282)
Supplement: Supplementary file 4 [file Table3.DOCX]

| **Supplementary Table III: Top 50 upregulate genes in DFSP** | | | |
| --- | --- | --- | --- |
| Gene name | Ensembl ID | Log2FoldChange | Padj |
| ADAM12 | ENSG00000148848 | 4.44396495 | 3.11E-54 |
| AFAP1 | ENSG00000196526 | 2.51365461 | 5.50E-46 |
| PDE10A | ENSG00000112541 | 5.34586731 | 1.09E-45 |
| FBN2 | ENSG00000138829 | 7.76044833 | 1.12E-44 |
| TRO | ENSG00000067445 | 3.32207988 | 1.16E-43 |
| CDH11 | ENSG00000140937 | 4.26053163 | 1.06E-39 |
| KIAA1755 | ENSG00000149633 | 4.31461454 | 1.95E-39 |
| GABRR1 | ENSG00000146276 | 9.50058675 | 3.00E-39 |
| CRACD | ENSG00000109265 | 5.72180201 | 5.19E-37 |
| CHST1 | ENSG00000175264 | 4.09582534 | 1.28E-34 |
| GASK1B | ENSG00000164125 | 2.6505013 | 2.13E-34 |
| COL26A1 | ENSG00000160963 | 7.43890734 | 1.69E-32 |
| CXCL13 | ENSG00000156234 | 9.43597645 | 3.42E-32 |
| IKBIP | ENSG00000166130 | 2.30619038 | 6.40E-32 |
| PTPRG | ENSG00000144724 | 2.66057659 | 1.65E-31 |
| ITGA11 | ENSG00000137809 | 4.07938508 | 2.03E-30 |
| PCDH18 | ENSG00000189184 | 4.36379252 | 5.70E-30 |
| DNAJC12 | ENSG00000108176 | 5.47004881 | 8.22E-30 |
| GPR137C | ENSG00000180998 | 3.31307538 | 6.91E-29 |
| C1orf61 | ENSG00000125462 | 5.50410642 | 1.32E-28 |
| PDE7B | ENSG00000171408 | 3.41222477 | 1.97E-27 |
| CAPN5 | ENSG00000149260 | 1.98165298 | 2.48E-27 |
| PEAK1 | ENSG00000173517 | 1.70192474 | 8.00E-27 |
| ALX4 | ENSG00000052850 | 5.86041005 | 4.26E-26 |
| TMEM59L | ENSG00000105696 | 6.0733415 | 4.73E-26 |
| LONRF2 | ENSG00000170500 | 3.95150635 | 7.21E-26 |
| OAF | ENSG00000184232 | 2.56900047 | 7.21E-26 |
| FAM20A | ENSG00000108950 | 3.67466236 | 7.57E-26 |
| CHST2 | ENSG00000175040 | 3.67292399 | 1.52E-25 |
| SPRY4 | ENSG00000187678 | 4.3385065 | 2.89E-25 |
| WDR86 | ENSG00000187260 | 3.66925991 | 3.49E-25 |
| GPR173 | ENSG00000184194 | 2.95582544 | 3.56E-25 |
| PPM1E | ENSG00000175175 | 5.67147538 | 3.68E-25 |
| RNF157 | ENSG00000141576 | 4.14505793 | 6.24E-25 |
| AFF3 | ENSG00000144218 | 3.47850723 | 1.09E-24 |
| BPI | ENSG00000101425 | 5.2037776 | 1.92E-24 |
| TMEFF1 | ENSG00000241697 | 3.90372622 | 2.33E-24 |
| SULF1 | ENSG00000137573 | 3.35823156 | 2.63E-24 |
| THY1 | ENSG00000154096 | 3.47169396 | 4.40E-24 |
| CAMK2A | ENSG00000070808 | 5.63350599 | 4.87E-24 |
| CHN1 | ENSG00000128656 | 4.38372139 | 5.05E-24 |
| CRMP1 | ENSG00000072832 | 2.69339527 | 5.80E-24 |
| GPX8 | ENSG00000164294 | 2.3068889 | 5.96E-24 |
| FHAD1 | ENSG00000142621 | 3.96960684 | 1.10E-23 |
| ADAMTS6 | ENSG00000049192 | 3.91032378 | 1.12E-23 |
| ASPN | ENSG00000106819 | 3.66705916 | 1.12E-23 |
| HHIPL1 | ENSG00000182218 | 2.24623774 | 1.65E-23 |
| SLC10A4 | ENSG00000145248 | 6.05147186 | 3.91E-23 |
| NRXN3 | ENSG00000021645 | 4.7604473 | 4.04E-23 |
| SV2A | ENSG00000159164 | 3.4662528 | 7.62E-23 |
